# The E3 ubiquitin ligase NEDD4 mediates cell migration signaling of EGFR in lung cancer cells

**DOI:** 10.1186/s12943-018-0784-2

**Published:** 2018-02-19

**Authors:** Genbao Shao, Ranran Wang, Aiqin Sun, Jing Wei, Ke Peng, Qian Dai, Wannian Yang, Qiong Lin

**Affiliations:** School of Medicine, Jiangsu University, Zhenjiang, Jiangsu 212013 China

**Keywords:** Lung cancer cell migration, EGFR, NEDD4, Lysosomal secretion, Cathepsin B

## Abstract

**Background:**

EGFR-dependent cell migration plays an important role in lung cancer progression. Our previous study observed that the HECT E3 ubiquitin ligase NEDD4 is significantly correlated with tumor metastasis and required for migration and invasion signaling of EGFR in gastric cancer cells. However, how NEDD4 promotes the EGFR-dependent lung cancer cell migration is unknown. This study is to elucidate the mechanism by which NEDD4 mediates the EGFR lung cancer migration signaling.

**Methods:**

Lentiviral vector-loaded *NEDD4* shRNA was used to deplete endogenous NEDD4 in lung cancer cell lines. Effects of the NEDD4 knockdown on the EGFR-dependent or independent lung cancer cell migration were determined using the wound-healing and transwell assays. Association of NEDD4 with activated EGFR was assayed by co-immunoprecipitation. Co-expression of NEDD4 with EGFR or PTEN was determined by immunohistochemical (IHC) staining in 63 lung adenocarcinoma tissue samples. Effects of NEDD4 ectopic expression or knockdown on PTEN ubiquitination and down-regulation, AKT activation and lysosomal secretion were examined using the GST-Uba pulldown assay, immunoblotting, immunofluorescent staining and a human cathepsin B ELISA assay respectively. The specific cathepsin B inhibitor CA-074Me was used for assessing the role of cathepsin B in lung cancer cell migration.

**Results:**

Knockdown of NEDD4 significantly reduced EGF-stimulated cell migration in non-small cell lung carcinoma (NSCLC) cells. Co-immunoprecipitation assay found that NEDD4 is associated with EGFR complex upon EGF stimulation, and IHC staining indicates that NEDD4 is co-expressed with EGFR in lung adenocarcinoma tumor tissues, suggesting that NEDD4 might mediate lung cancer cell migration by interaction with the EGFR signaling complex. Interestingly, NEDD4 promotes the EGF-induced cathepsin B secretion, possibly through lysosomal exocytosis, as overexpression of the ligase-dead mutant of NEDD4 impedes lysosomal secretion, and knockdown of NEDD4 significantly reduced extracellular amount of cathepsin B induced by EGF. Consistent with the role of NEDD4, cathepsin B is pivotal for both basal and the EGF-stimulated lung cancer cell migration. Our studies propose a novel mechanism underlying the EGFR-promoted lung cancer cell migration that is mediated by NEDD4 through regulation of cathepsin B secretion.

**Conclusion:**

NEDD4 mediates the EGFR lung cancer cell migration signaling through promoting lysosomal secretion of cathepsin B.

## Background

NEDD4 (also NEDD4–1) is a member of the HECT E3 ubiquitin ligase family and initially found in regulation of the proteasomal degradation of epithelial sodium channel (ENaC) [1]. Defect in ubiquitination of ENaC by NEDD4 causes the hypertension disease Liddle Syndrome [2]. Now studies have shown that NEDD4 has many ubiquitination substrates via interaction with its four WW domains [3], and plays important roles in multiple cellular functions [4]. It has been observed that the NEDD4 yeast homologue Rsp5p is required for membrane protein endocytosis and transport to vacuoles [5], and involved in regulating ubiquitination-mediated multivesicular body (MVB) sorting process [6]. In mammalian cells, NEDD4 is involved in endosomal trafficking of receptor tyrosine kinases EGFR and FGFR by ubiquitination of endocytic or vesicle sorting proteins, such as Cbl, Eps15, Tsg101, Hrs, SCAMPs, and ACK1 [7–13]. Knockdown of NEDD4 in A549 cells inhibited the ligand-induced endosomal trafficking and lysosomal degradation of EGFR, and significantly elevated the expression level of EGFR [13]. Our recent studies have shown that NEDD4 directly interacts with autophagic protein LC3B via its LC3-interactive region (LIR) and ubiquitinates SQSTM, a key player in selective autophagy [14, 15]. Knockdown of NEDD4 caused defect in autophagy, accumulation of autophagosomes in endoplasmic reticulum (ER) and formation of protein inclusion bodies [14, 15], suggesting that NEDD4 plays an important role in selective autophagy.

Recent studies indicate that NEDD4 is involved in tumorigenesis and progression. Overexpression of NEDD4 has been found in multiple types of solid tumors [16]. NEDD4 was reported to interact with, ubiquitinate and down-regulate PTEN, a tumor suppressor [17]. In addition, NEDD4 mono-ubiquitinates and translocates PTEN from cytoplasm to nuclei [18]. Nuclear translocation might be required for the tumor suppressing activity of PTEN [18], presumably through maintaining chromosomal integrity and genomic stability [19]. Our studies have shown that NEDD4 overexpressed in gastric cardia adenocarcinoma (GCA), and its overexpression is correlated with the tumor invasion and metastasis, and inversely associated with the survival rate [20]. The 5-year survival rate in NEDD4-negative GCA patients is as high as 96% [20], suggesting that NEDD4 is an oncogenic protein that plays a key role in GCA tumor progression and metastasis.

EGFR, a member of the HER receptor tyrosine kinase family, is a known oncogenic protein in solid tumors, particularly in lung cancer [21]. Inhibitors of EGFR have been used for targeted therapy clinically [22, 23]. Many studies have shown that overexpression of EGFR is associated with tumor invasion, metastasis, and relapse in multiple types of cancers [24–27]. EGFR promotes cell migration and invasion signaling in cancer cells through activation of cell adhesion, SRC, AKT, MAPK and endosomal signaling pathways [28–33]. Our previous studies observed that EGFR signaling activates the E3 ubiquitin ligase activity of NEDD4 [34]. Knockdown of NEDD4 severely impaired EGF-stimulated gastric cancer cell migration and invasion [20], suggesting that NEDD4 mediates migration and invasion signaling of EGFR. However, how NEDD4 mediates the EGFR-dependent cancer cell migration remains elusive.

In this research article, we demonstrated that NEDD4 interacts with EGFR upon EGF stimulation in lung cancer cells. Knockdown of NEDD4 significantly reduces EGFR-promoted lung cancer cell migration rate. Furthermore, knockdown of NEDD4 inhibits EGF-dependent unconventional lysosomal cathepsin B secretion, which is an important cellular process for lung cancer cell migration. Our studies have revealed a new EGFR migration signaling pathway that is mediated by NEDD4 and cathepsin B secretion.

## Results

### NEDD4 is required for the EGF-promoted lung cancer cell migration.

**Fig. 1:**
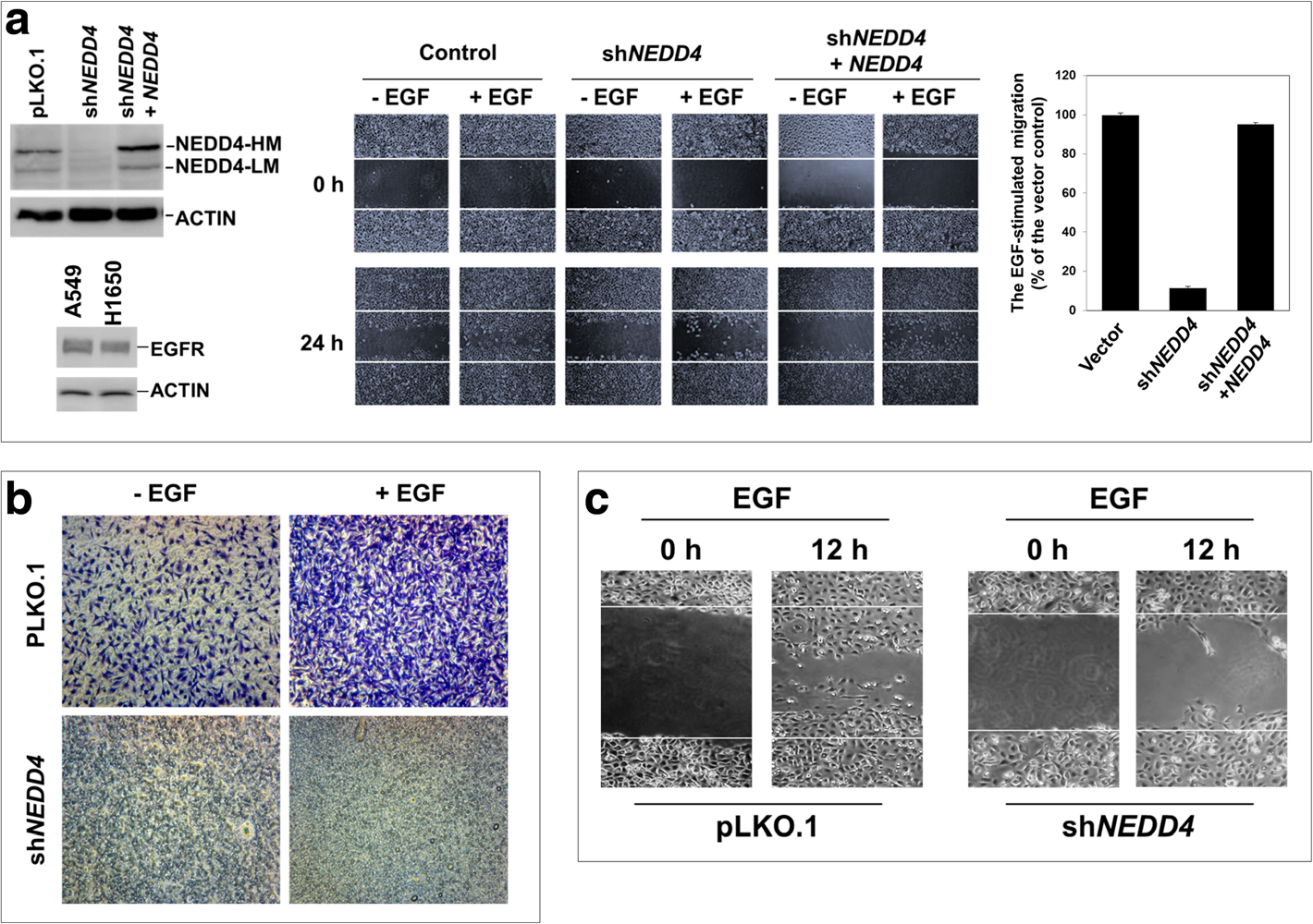
NEDD4 mediates EGFR-dependent lung cancer cell migration. **a**, Wound healing assay of A549 cell migration. Left top panel, the knockdown of NEDD4 by shNEDD4 (lane 2) and recovery of NEDD4 upon re-introducing NEDD4 cDNA in the knockdown cells (lane 3); NEDD4-HM, high molecular weight NEDD4; NEDD4-LM, low molecular weight NEDD4. Left bottom panel, the protein level of EGFR in the lung cancer cell lines A549 and H1650 shown by immunoblotting with the cell lysates. Middle panel, photo images of the cell migration. Right panel, quantification of the EGF-stimulated cell migration area occupied after 24 h from the data of three independent experiments using the imaging software Image J (NIH). The non-EGF-treated cell migration area was subtracted by the EGF-treated cell migration area to obtain the EGF-stimulated cell migration area. **b**, Transwell assay of A549 cell migration. Note that the small lightly-stained round dots are pores of the transwell plates (sh*NEDD4* panels). **c**, Wound healing assay of H1650 cells

EGFR mutation is a key driving factor for tumorigenesis and progression in non-small cell lung cancer (NSCLC) [35, 36]. As our previous studies have shown that NEDD4 regulates EGFR endosomal trafficking for lysosomal degradation in NSCLC cells [13], and NEDD4 mediates EGF-promoted migration and invasion in gastric cancer cells [20], we wonder if NEDD4 plays the same role in mediating the EGFR migration signaling in NSCLC cells as in gastric cancer cells. NEDD4 was depleted by lentiviral vector-loaded *NEDD4*-shRNA (sh*NEDD4*) in two NSCLC cell lines A549 and H1650 (Fig. 1). A549 cells express wild type EGFR and H1650 cells contain a kinase domain deletion mutation of EGFR [37]. Notice that in the left panel of Fig.1A NEDD4-HM stands for high molecular weight NEDD4, which is the full length of NEDD4, while NEDD4-LM for low molecular weight NEDD4, which is a degradation product of NEDD4-HM [14]. As shown in Fig. 1A, *shNEDD4* depleted more than 90% of NEDD4 in A549 cells (the left panel) and impaired EGF-stimulated cell migration in a wound-healing assay (the middle panel), and inhibited about 90% of the migration rate (the right top panel). Furthermore, re-expression of the shRNA-resistant NEDD4 in the knockdown cells recovered cell migration capacity. These data suggest that NEDD4 mediates the EGFR migration signaling in lung cancer A549 cells.

To confirm the role of NEDD4 in the EGFR migration signaling, we carried out a transwell assay for detection of the NEDD4 knockdown effect on migration of A549 cells. As shown in Fig. 1B, knockdown of NEDD4 diminished both the EGF- and the non-EGF-dependent cell migration capacity evaluated by penetration of micro-pores of the membrane in the transwell, which resembles the escaping process of tumor cells from tumor tissues into blood stream. This data indicates that NEDD4 is not only involved in the EGF-dependent, but also in the non-EGF dependent cell migration in A549 cells. Furthermore, we examined the role of NEDD4 in lung cancer H1650 cells that contain an EGFR deletion mutation, which is a common mutation that drives tumorigenesis and progression in lung cancer patients [35]. Consistent with the results in A549 cells, knockdown of NEDD4 in H1650 cells eliminated the cell migration capacity (Fig. 1C). Taken together, our data have demonstrated that NEDD4 is a key E3 ubiquitin ligase mediating the EGFR cell migration signaling in lung cancer cells.

### NEDD4 interacts with EGFR in lung cancer cells.

**Fig. 2:**
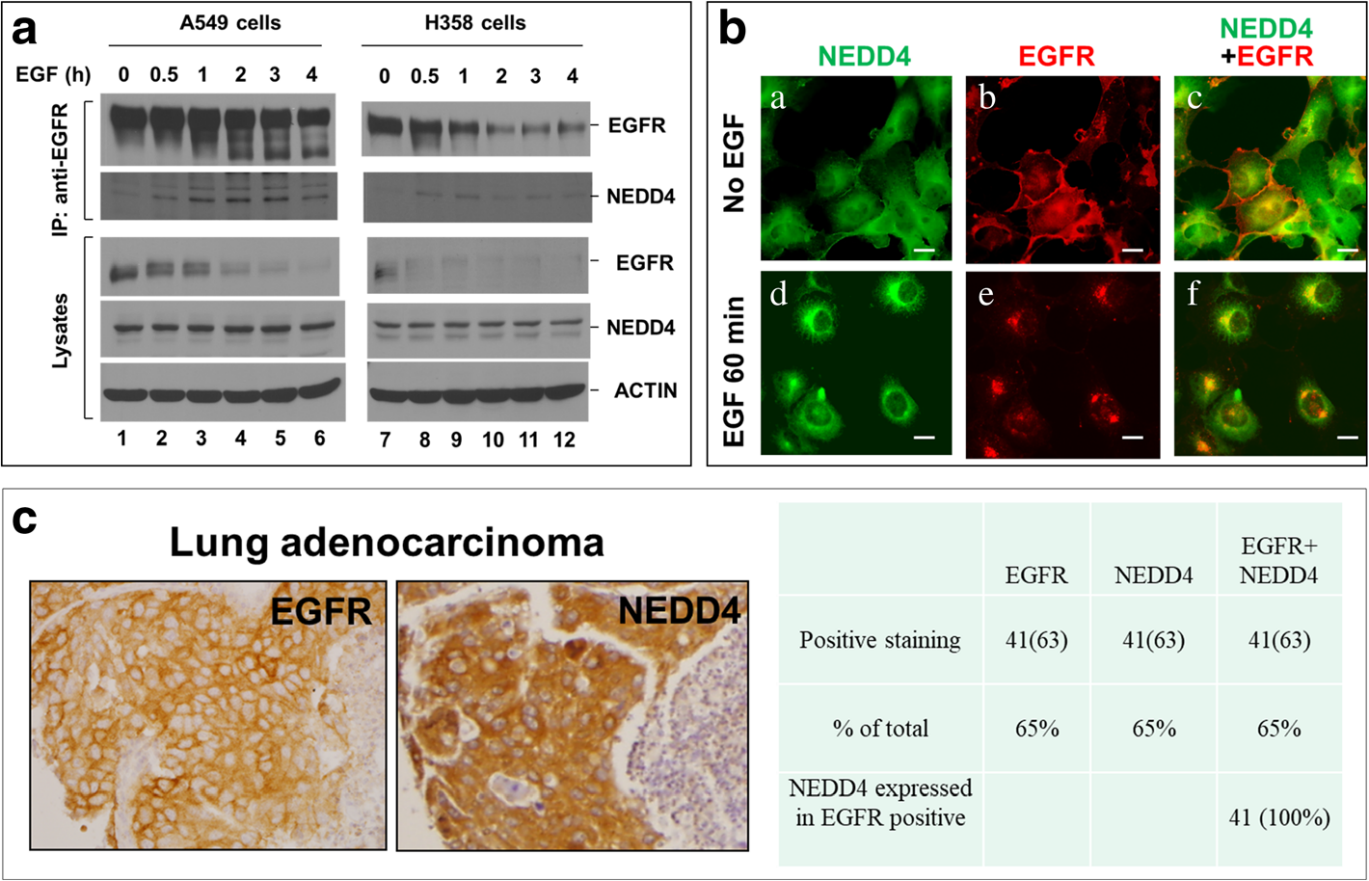
NEDD4 is associated with activated EGFR. **a**, Co-immunoprecipitation of NEDD4 with activated EGFR in lung cancer cells. Lung cancer A549 or H358 cells were serum-starved for 12 h followed by stimulation with EGF (50 ng/ml) for indicated times. EGFR was immunoprecipitated with anti-EGFR (Mab528) and detected by immunoblotting with anti-EGFR (1005) (top panels). Co-immunoprecipitated NEDD4 was detected by immunoblotting with an anti-NEDD4 (second top panels). The level of EGFR and NEDD4 in the cell lysates was also detected by immunoblotting (middle and second bottom panels). Notice that EGFR in A549 and H358 cells has an EGF-induced degradation. **b**, Internalized EGFR is co-localized with NEDD4. A549 cells were serum-starved for 12 h followed by stimulation with EGF (50 ng/ml) for 0 or 60 min. The cells were immuno-stained with anti-EGFR (1005) (red) and anti-NEDD4 (green). Bar, 20 μM. **c**, Co-expression of NEDD4 with EGFR in lung adenocarcinoma tissue. The tissue microarray containing 63 lung adenocarcinoma section samples was immunohistochemically stained with anti-EGFR or anti-NEDD4

To further investigate the mechanism underlying the effect of NEDD4 on EGF-stimulated lung cancer cell migration, we first examined whether NEDD4 is in the EGFR signaling complex. Lung cancer A549 or H358 cells were stimulated with EGFR for 0–4 h (Fig. 2A). EGFR was immunoprecipitated from the lysates with an anti-EGFR (Mab528), and the co-immunoprecipitated NEDD4 was detected by immunoblotting with an anti-NEDD4. As shown in Fig. 2A, NEDD4 was co-immunoprecipitated with EGFR upon EGF stimulation in both A549 and H358 cells, suggesting that NEDD4 specifically interacts with activated EGFR in lung cancer cells. Notice that EGFR in both cell lines has ligand induced degradation and NEDD4 is specifically associated with the activated EGFR complex, which is consistent with our previous findings about the role of NEDD4 in regulating endosomal trafficking and lysosomal degradation of EGFR through interaction with and ubiquitination of ACK1, an EGFR-binding protein [13]. As ACK1 is co-localized with EGFR on endosomes [38], we suspected that NEDD4 might be also co-localized with EGFR on endosomes. As expected, the immunofluorescent staining of endogenous NEDD4 and EGFR in A549 cells upon stimulation with EGF has shown that NEDD4 is specifically co-localized with the internalized EGFR (Fig. 2B), suggesting that NEDD4 interacts with EGFR on endosomes.

To address whether NEDD4 is co-expressed with EGFR in lung tumor tissues, we immuno-stained 63 lung adenocarcinoma tumor tissue samples with both anti-NEDD4 and anti-EGFR in a tissue microarray (TMA) assay. As shown in Fig. 2C, both NEDD4 and EGFR are overexpressed in 41 lung adenocarcinoma samples out of total 63 samples, both overexpression rate in lung adenocarcinoma tumors are 65%. More importantly, NEDD4 and EGFR are always co-expressed in lung adenocarcinoma tumor tissue (the right panel, Fig. 2C), suggesting that NEDD4 might be associated with EGFR in lung adenocarcinoma.

### The NEDD4-mediated EGFR migration signaling is not dependent on the PTEN/PI3K/AKT pathway in lung cancer cells.

**Fig. 3:**
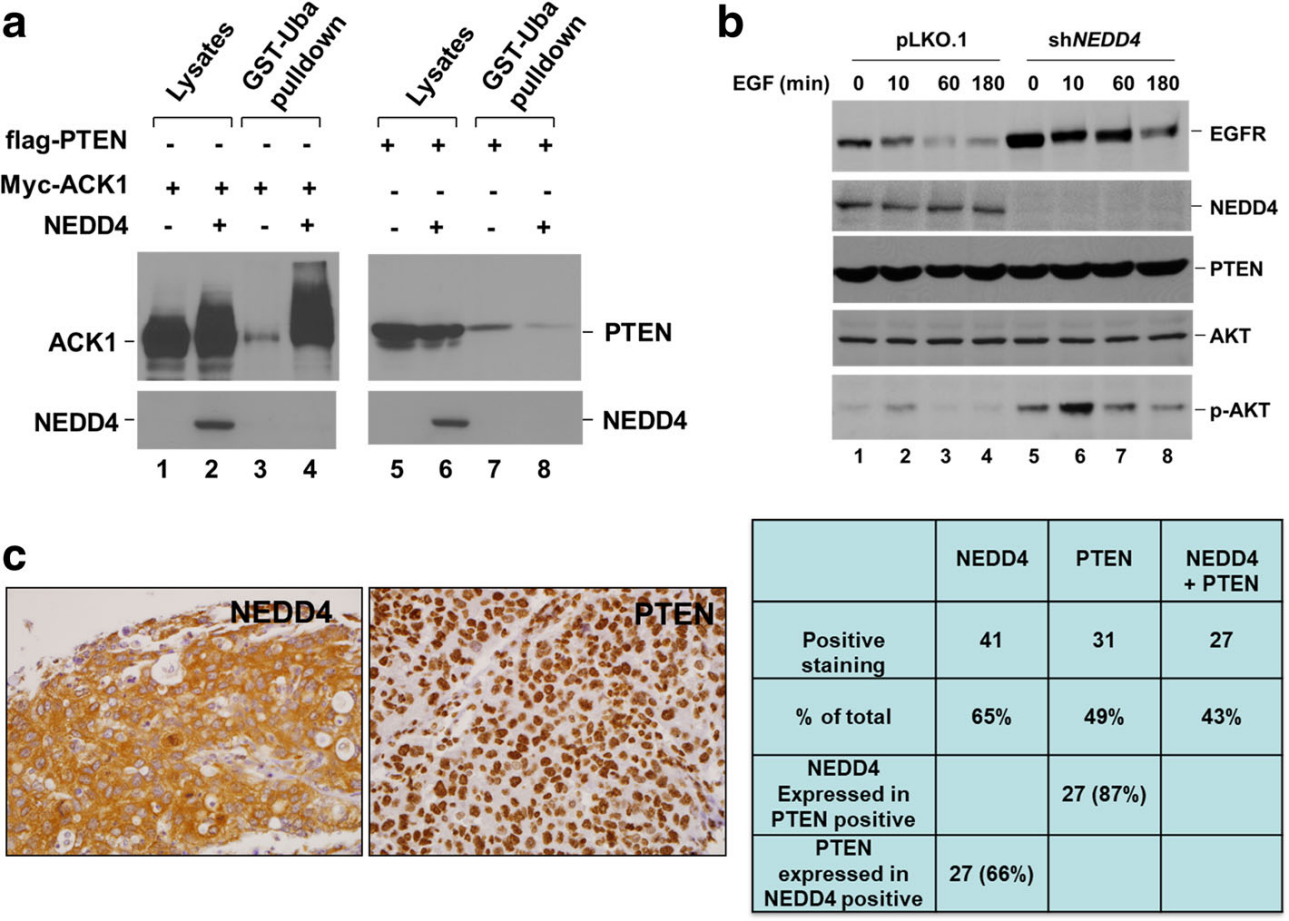
NEDD4 does not ubiquitinate and downregulate PTEN. **a**, NEDD4 was co-expressed with flag-PTEN or Myc-ACK1 by transfection in HEK293 cells. Ubiquitinated ACK1 or PTEN was precipitated with bead-bound GST-Uba from the cell lysates followed by immunoblotting with anti-Myc or anti-flag antibodies. **b**, Lung cancer A549 cells were infected with lenti-viral vector pLKO.1 or the vector-loaded sh*NEDD4*. NEDD4 in the cell lysates was detected by immunoblotting with anti-NEDD4 (second top panel). The effect of knockdown NEDD4 on expression of PTEN and activation of AKT was assessed by immunoblotting PTEN AKT or phospho-AKT in the cell lysates with their antibodies respectively. **c**, Immunohistochemical (IHC) staining of 63 human lung adenocarcinoma tumors with anti-NEDD4 and anti-PTEN antibodies. The positive tumor samples were assessed and counted under microscope and listed in the table

Numbers of research reports have observed that NEDD4 regulates cancer cell proliferation through ubiquitination and down-regulation of the tumor suppressor PTEN [16, 39–41], which is an inhibitor of the PI3K/AKT pathway. The PI3K/AKT pathway is known to promote cancer cell survival and migration [42, 43]. However, there are discrepant conclusions about the role of NEDD4 in ubiquitination and degradation of PTEN in previous studies [44, 45]. Thus, we examined whether NEDD4 ubiquitinates and down-regulates PTEN and activates the PI3K/AKT pathway. To determine the ubiquitination, flag-tagged PTEN was co-expressed with NEDD4 in HEK293 cells (Fig. 3A). At the same time, we used ACK1, a known NEDD4 substrate [13], as a positive control for the ubiquitination. Poly-ubiquitinated proteins were precipitated with GST-Uba and detected by immunoblotting with indicated antibodies (Fig. 3A). While ACK1 was heavily poly-ubiquitinated by NEDD4 (lane 4, left panel), PTEN was not poly-ubiquitinated by NEDD4 (lane 8, right panel), indicating that PTEN is not a poly-ubiquitinated substrate of NEDD4 under the condition.

We further examined if knockdown of NEDD4 inactivates AKT. NEDD4 was depleted by lentiviral vector-loaded sh*NEDD4* in A549 cells, and the cells were stimulated by EGF at indicated time (Fig. 3B). PTEN and phospho-AKT (S473) in the cell lysates were detected by immunoblotting. Upon knockdown of NEDD4, PTEN protein level had no observable change (lanes 5–8, Fig. 3B), while phospho-AKT (S473) markedly increased, probably due to an increase of EGFR level resulting from impairment of the degradation, as we reported previously [13]. Furthermore, depletion of NEDD4 did not impede activation of AKT by EGFR signaling (lane 6, Fig. 3B). These data suggest that NEDD4 is not an upstream protein for AKT activation, and its promoting effect on the lung cancer cell migration is unlikely to be mediated by the PTEN/PI3K/AKT pathway.

To determine whether expression of NEDD4 has an opposite pattern to that of PTEN in lung adenocarcinoma tissue samples, 63 lung adenocarcinoma samples were immuno-stained with both anti-NEDD4 and anti-PTEN using a tissue microarray assay (TMA). As shown in Fig. 3C, NEDD4 is co-expressed with PTEN in 27 samples out of total 31 PTEN-positive samples, or 87% of the PTEN-positive samples; while PTEN is co-expressed with 27 samples out of 41 NEDD4-positive samples, or 66% of the NEDD4-positive samples. Interestingly, the PTEN staining is exclusively in nuclei (Fig. 3C). These data indicate that expression of PTEN is not reversely correlated with that of NEDD4, suggesting NEDD4 might not poly-ubiquitinate and down-regulate PTEN in lung adenocarcinoma tumors.

Taken together, we conclude that it is unlikely for NEDD4 to promote lung cancer cell migration through ubiquitination and down-regulation of PTEN.

### NEDD4 is required for the EGF-stimulated unconventional lysosomal secretion.

**Fig. 4:**
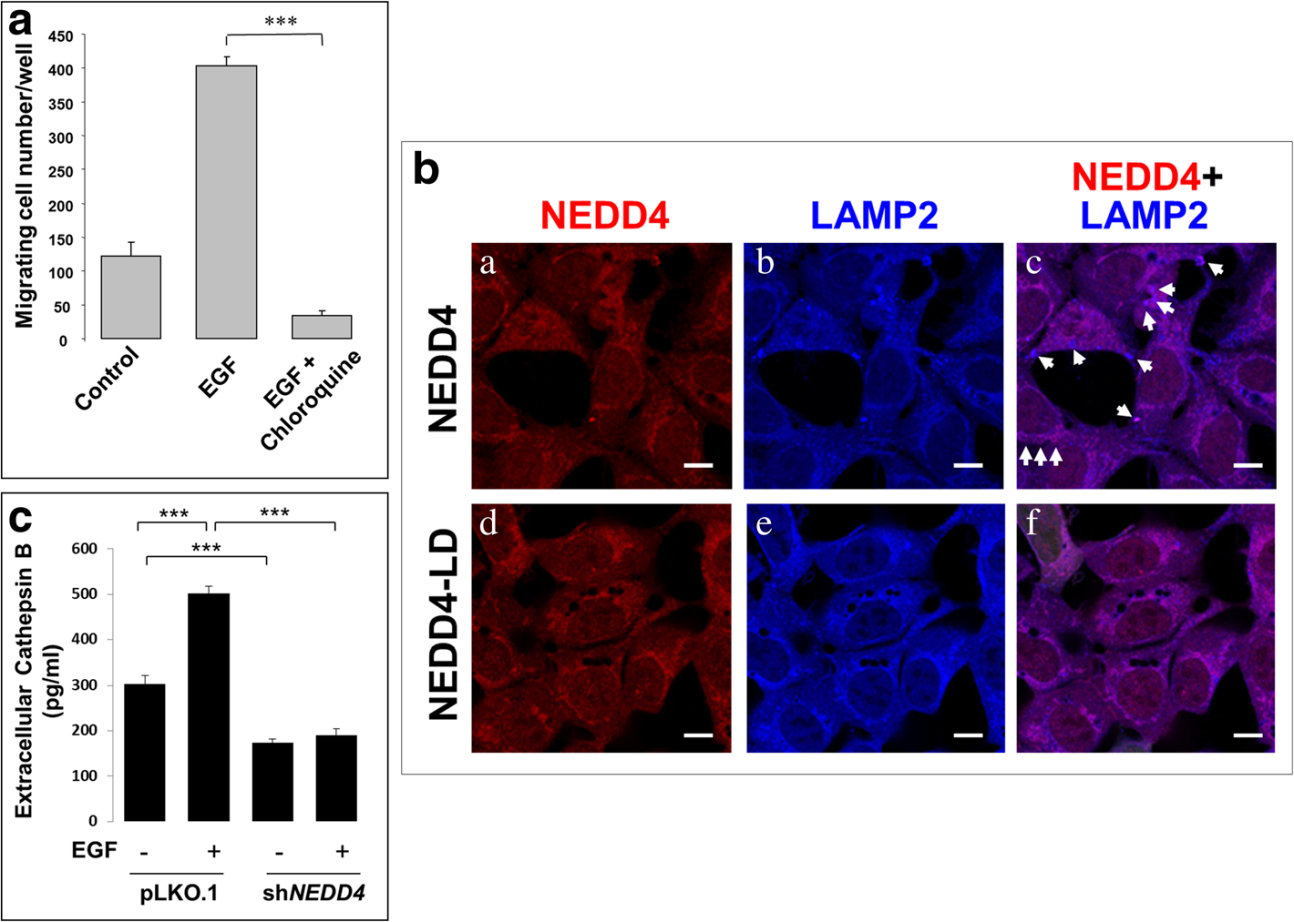
NEDD4 is required for EGF-stimulated lysosomal secretion of cathepsin B. **a**, Lysosomes function in lung cancer cell migration. A549 cells were resuspended in serum-free medium and used for transwell cell migration assay. The migration attractant was 10% fetal bovine serum plus or minus EGF (50 ng/ml). The lysosome inhibitors chloroquine (10 μM) was added in the medium with EGF. The cells migrated from the top well to the bottom well within 6 h. The migrated cells were stained and quantified as described in the section of Methods. **b**, Overexpression of the NEDD4 ligase-dead mutant NEDD4[C867A] eliminated the LAMP2-positive vesicles at cell edges. NEDD4 or the ligase-dead mutant was stably expressed in A549 cells. The cells were stimulated with EGF (50 ng/ml) for 30 min, followed by immunofluorescent staining. NEDD4 and LAMP2 were stained with anti-NEDD4 and anti-LAMP2. The white arrows indicate the putative lysosomal secretion vesicles. NEDD4-LD stands for the ligase-dead mutant of NEDD4, NEDD4[C867A]. Bar, 20 μM. **c**, The culture medium collected from the vector control or shNEDD4 cells treated with or without EGF for 12 h was used for detection of cathepsin B with a human cathepsin B ELISA assay kit. The assay was repeated three times. ***, *p* < 0.001

It has been observed that the EGFR-dependent cell migration requires an endosomal/vesicle transport process [46]. Our previous studies observed that NEDD4 regulates the EGFR endosomal trafficking and lysosomal degradation [13]. We wonder if the role of NEDD4 in endosomal trafficking is relevant to the EGFR-promoted lung cancer cell migration. So we first tested the effect of the lysosomal inhibitor chloroquine on the EGF-stimulated migration of lung cancer A549 cells using a transwell assay. As shown in Fig. 4A, while EGF induced a significant enhancement of migration cell numbers, treatment with chloroquine diminished the EGF-dependent lung cancer cell migration. This piece of data suggests that lysosomal function is required for the EGF-dependent lung cancer A549 cell migration.

Early studies have shown that unconventional secretion of lysosomes is involved in cancer metastasis and cell migration and invasion [47–49], and NEDD4 participates in the ESCRT-dependent viral budding process, which resembles the MVB-dependent or the unconventional lysosomal secretion [50, 51]. In addition, Rsp5p, the yeast homologue of NEDD4, directly regulates the ubiquitination-dependent sorting process of MVBs [6]. These studies lead us to hypothesize that NEDD4 regulates lung cancer cell migration through the unconventional lysosomal secretion. To test this hypothesis, we ectopically overexpressed NEDD4 or its ligase-dead mutant NEDD4 [C867A] in A549 cells using a lenti-viral expression system. By staining the lysosomal marker LAMP2, we observed that numbers of the LAMP2-positive vesicles appeared at the cell edges in the NEDD4-overexpressed cells upon EGF stimulation for 30 min (as indicated with white arrows in Fig. 4B). Some of these LAPM2-positive vesicles were co-stained with NEDD4 (Fig. 4B). However, when the ligase-dead mutant of NEDD4 (labeled as NEDD4-LD in Fig. 4B) was overexpressed, no LAMP2-positive vesicle structure was observed at the cell edges (Fig. 4B). These data suggest that NEDD4 ligase activity might promote lysosomal secretion.

To confirm the role of NEDD4 in lysosomal secretion, we detected the secreted lysosomal protease cathepsin B in culture medium using an ELISA assay in both the vector control and the sh*NEDD4* lung cancer A549 cells with or without EGF stimulation. As shown in Fig. 4C, in the vector control cells, EGF dramatically stimulated cathepsin B secretion. While in the NEDD4 knockdown (sh*NEDD4*) cells the basal (non-EGF) level of the secreted cathepsin B was dropped about 50%, and the EGF-stimulated secretion of cathepsin B was eliminated. These data indicate that both the EGF-dependent and the non-EGF-dependent lysosomal secretion (cathepsin B) require NEDD4.

### The lysosomal protease cathepsin B is important for both the EGF and the non-EGF dependent lung cancer cell migration.

**Fig. 5:**
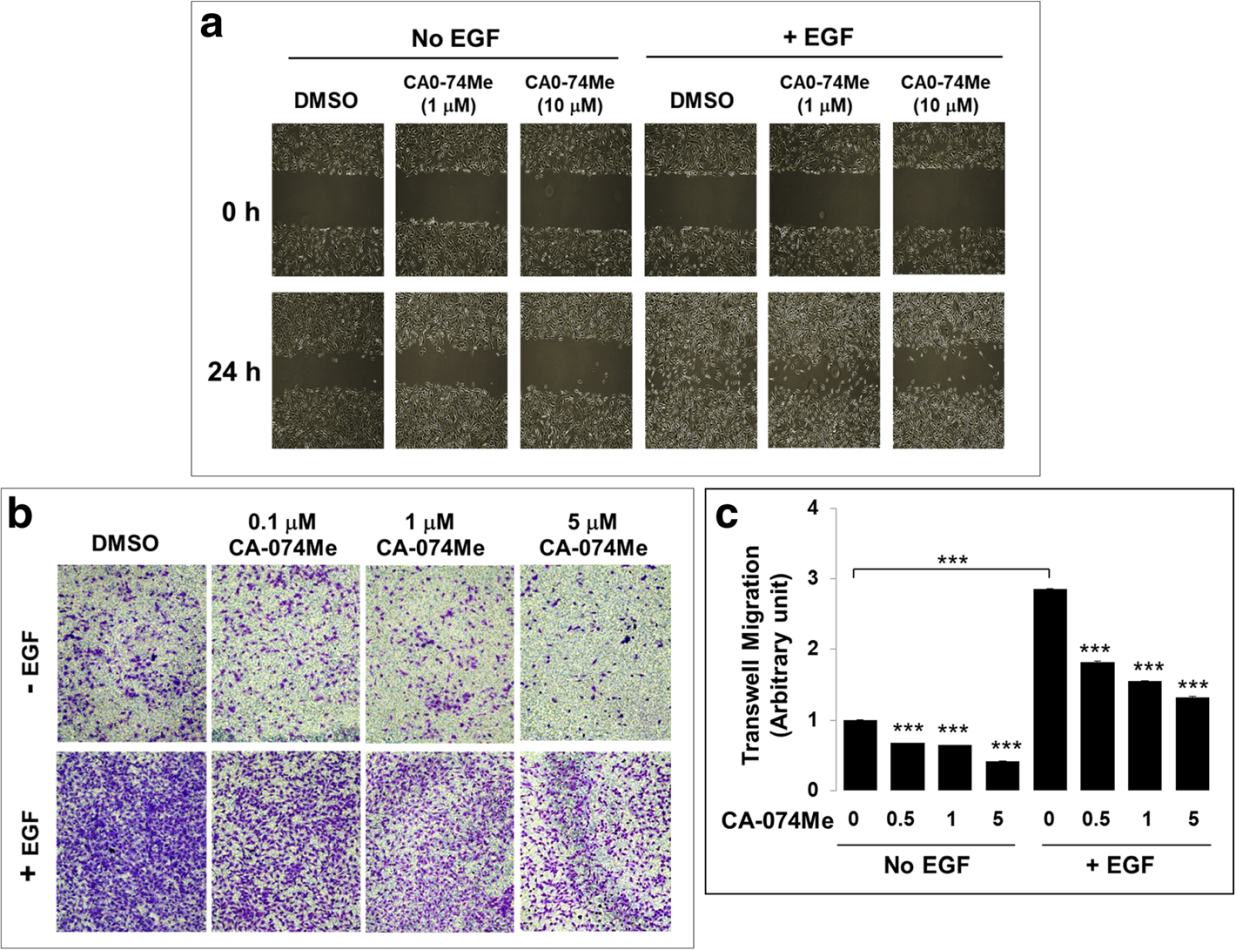
Cathepsin B plays an important role in lung cancer cell migration. **a**, The effect of the cathepsin B inhibitor CA-074Me on the EGF-stimulated lung cancer A549 cell migration determined by the wound healing assay. **b**, The effect of the cathepsin B inhibitor CA-074Me on the EGF-stimulated lung cancer A549 cell migration determined by the transwell assay. **c**, Quantification of the data from three independent transwell migration experiments. The statistics was performed with the treatment sample vs its control. ***, *p* < 0.001

To connect the NEDD4-mediated lung cancer cell migration, including both the EGF and the non-EGF dependent lung cancer cell migration, to lysosomal secretion, we examined the effect of CA-074Me, a specific inhibitor of cathepsin B, on lung cancer A549 cell migration using a wound healing assay (Fig. 5A). Treatment of the cells with 10 μM CA-074Me significantly inhibited both the non-EGF-dependent (basal) and the EGF-stimulated lung cancer cell migration (Fig. 5A). To confirm the effect, we also used a transwell assay to detect the effect of CA-074Me on A549 cell migration. As shown in Fig 5B and C, treatment with 5 μM CA-074Me diminished more than 60% of the non-EGF-dependent cell migration and more than 80% of the EGF-dependent cell migration. These data indicate that cathepsin B has a significant role in A549 cell migration, and strongly suggest that NEDD4 mediates the EGFR lung cancer cell migration through the lysosomal secretion pathway.

## Discussion

Our previous studies observed that NEDD4 is overexpressed in gastric cardia carcinoma and significantly correlated with both local and remote metastasis and reversely associated with patient’s survival [20]. Knockdown of NEDD4 in gastric cancer cells severely impaired cell migration and invasion [20]. Furthermore, NEDD4 interacts with and ubiquitinates ACK1, which is an EGFR binding protein, and regulates EGFR endosomal trafficking and lysosomal degradation [13]. In this report, we found that NEDD4 interacts with EGFR and participates in both the basal and the EGFR-signaling-dependent lung cancer cell migration. Immunohistochemical (IHC) staining of lung adenocarcinoma indicates that NEDD4 is co-expressed with EGFR. More importantly, NEDD4 mediates both the EGFR-dependent and -independent secretion of lysosomal cathepsin B, which in turn promotes lung cancer cell migration. Our studies have provided a new vision to the mechanism underlying the NEDD4-mediated lung cancer cell migration.

Cathepsin B has been established as a biomarker for tumor angiogenesis and metastasis [52–54]. Expression of cathepsin B has been associated with tumor invasiveness and metastasis in multiple types of cancer [52–54]. The mechanism underlying the promoting effect of cathepsin B on cancer cell migration and invasion has been investigated. It has been reported that cathepsin B promotes cancer cell migration or invasion through proteolysis of extracellular matrix [55] and activation of Toll-like receptor 3 (TLR3) [56] and uPA [57]. One of the studies observed that lysosomal secretion of cathepsin B to podosomal sites to degrade extracellular focal matrix that promotes the podosome-dependent cell migration and invasion [58], indicating a direct role of lysosomal secretion in facilitating cell migration and invasion. Cathepsin B also mediates the interleukine 8 (IL-8)/CXCR2-activated endothelial cell migration through cleavage of HB-EGF and activation of EGFR [59]. These data strongly support that lysosomal secreted cathepsin B route is an important pathway for promoting cell migration and invasion in both cancer and endothelial cells.

Our studies observed no significant ubiquitination and down-regulation of PTEN by either overexpression or knockdown of NEDD4 (Fig. 3). Furthermore, knockdown of NEDD4 increased amount of the active phospho-AKT (S473) upon EGF stimulation (Fig. 3B), probably due to the enhanced level of EGFR resulted from impairment of lysosomal degradation upon depletion of NEDD4 [13]. Dephosphorylation of pS473 of AKT has been demonstrated specifically sensitive to PTEN [60]. These data strongly suggest that the effect of NEDD4 on lung cancer cell migration is unlikely through ubiquitination and down-regulation of PTEN and activation of AKT. However, a recent report has shown that NEDD4 promotes hepatocellular carcinoma cancer cell migration through regulating the PI3K/AKT signaling by down-regulation of PTEN [61]. This discrepancy is possibly due to different cell systems, or different isoforms of NEDD4 in the studies. In fact, it has been shown that NEDD4 is dispensable for ubiquitination and down-regulation of PTEN [44], and that another HECT E3 ubiquitin ligase WWP2, not NEDD4, interacts with, ubiquitinates and down-regulates PTEN [45]. One recent report has shown that NEDD4 is a down-stream target of PI3K/AKT/mTORC1, rather than an up-stream ubiquitin ligase for degradation of PTEN [62]. Our data in Fig. 3B showing that depletion of NEDD4 does not impede activation of AKT by EGFR also suggest that NEDD4 is not an upstream component of the PI3K/AKT signaling. In addition, our immunohistochemical staining did not find a negative correlation between expression of NEDD4 and PTEN in lung adenocarcinoma tissue samples (Fig. 3C), which is inconsistent with a similar study on non-small-cell lung carcinoma tumor tissues [39]. The controversial results might be produced by difference in staining procedures, or the antibodies used in the staining. Further investigation is necessary to reconcile the discrepancy and clarify the exact role of NEDD4 in the PTEN/PI3K/AKT signaling pathway.

**Fig. 6:**
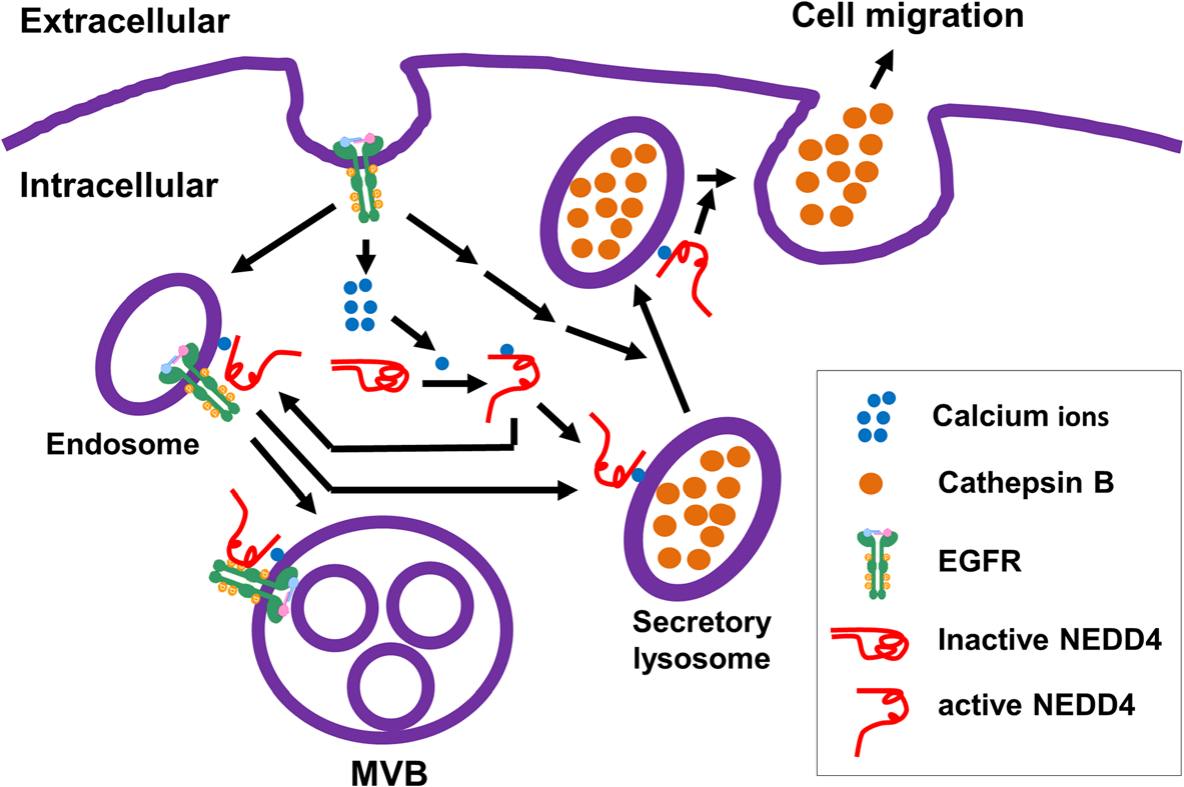
A proposed pathway of the NEDD4-mediated EGFR-dependent cell migration. Activated EGFR signaling elevates cytoplasmic calcium level and subsequently activates NEDD4. The activated NEDD4 is recruited to the EGFR endosomal complex and the secretary lysosomal vesicles, where NEDD4 interacts with and ubiquitinates the ESCRT complex to facilitate the engulfment of EGFR into MVB and the secretion of lysosmal cathepsin B to extracellular matrix. The secreted lysosomal cathepsin B hydrolyzes cell matrix/junction proteins and promotes cell migration

EGFR migration signaling in cancer cells has been extensively investigated, and several pathways, such as cell adhesion, Src, Akt, MAPK and endosomal signaling pathways [28–33], have been identified. The findings presented here combined with our previous studies on NEDD4 lead us to propose a novel EGFR lung cancer cell migration pathway mediated by NEDD4 via promoting secretion of cathepsin B, as depicted in Fig. 6. EGFR activation releases calcium from the ER pool and subsequently activates NEDD4 [34]. Activated NEDD4 then is recruited to the EGFR-loaded endosomes (Fig. 2B) or the ALIX/ESCRT vesicle transport machinery, as previous studies have shown [6, 50, 51]. On one hand, NEDD4 is collaborated with TNK2 (ACK1) to regulate transport of the EGFR-loaded endosomes to MVBs/lysosomes [13, 38]. On the other hand, the activated NEDD4 interacting with the endosomal EGFR or/and other signal proteins stimulates secretion of the lysosomal cathepsin B, most likely through regulation of the ESCRT complex for the membrane fusion between secretory lysosomes and plasma membrane, which resembles the process of viral budding mediated by NEDD4 [50, 51]. In this proposed pathway, the NEDD4-dependent secretion of the lysosomal cathepsin B is a key step for lung cancer cell migration. It should be pointed out that NEDD4 is activated not only by the EGFR signal but also by other signals, such as the G-protein-coupled receptor (GPCR) signal [34]. Thus, NEDD4 mediates both the EGFR-dependent and independent secretion of the lysosomal cathepsin B and cell migration, as we have shown in Fig. 1B and Fig. 4C.

However, the exact molecular mechanism by which NEDD4 promotes unconventional secretion of lysosomal cathepsin B currently remains unknown. There are two possible mechanisms: One is via regulation of the ALIX/ESCRT machinery to facilitate the secretory vesicle fusion with plasma membrane, which is similar to the process of viral budding regulated by NEDD4, as mentioned above. The other is via biogenesis and trafficking of autophagosomes. Our recent studies found that NEDD4 interacts with autophagosomal protein LC3, ubiquitinates autophagy receptor SQSTM1 and plays an important role in biogenesis and trafficking of autophagosomes [14, 15]. It has been demonstrated that autophagosomes are involved in unconventional secretion [63]. Thus, NEDD4 might promote lysosomal secretion of cathepsin B through facilitating formation and trafficking of autophagosomes. However, this autophagosome-involved secretion of lysosomal cathepsin B mediated by NEDD4 has not been explored to date. Further studies on these pathways in future are necessary for elucidation of the mechanism by which NEDD4 promotes secretion of cathepsin B and the lung cancer cell migration.

## Conclusions

(1) NEDD4 mediates the EGF-stimulated lung cancer cell migration; (2) NEDD4 does not ubiquitinate and down-regulate of PTEN and activate the PI3K/AKT pathway; (3) NEDD4 facilitates the EGFR-dependent lysosomal secretion of cathepsin B; (4) cathepsin B mediates lung cancer cell migration. Thus, it is likely that NEDD4 mediates the EGFR cell migration signaling in lung cancer cell lines through activation of the lysosomal cathepsin B secretion pathway.

## Methods

### Materials

Anti-NEDD4 was purchased from Millipore (07–049); anti-EGFR (1005) and anti-ACK1 (A11) was from Santa Cruz; anti-PTEN (#9552), anti-AKT (#9272) and anti-phospho-AKT (S473) (#9271) from Cell Signaling; anti-GFP (MMS-118R), anti-HA (MMS-101R) from BioLegend; anti-flag (M2) (F1804) and anti-ACTIN (A5441) from Sigma-Aldrich; anti-EGFR (Mab528) was prepared from culture medium of the EGFR (Mab528) hybridoma cell line (ATCC). The cathepsin B ELISA assay kit was from RayBiotech. Fluorescent dye-conjugated secondary antibodies and phalloidin were purchased from ThermoFisher. The cathepsin B inhibitor CA-074Me was purchased from Apexbio. The NEDD4 shRNA (5’-AUUUGAACCGUAUAGUUCAGC-3′) in the lentiviral expression vector pLKO.1 was purchased from Open Biosystems (RHS4533-EG4734). The lung cancer cell lines A549 and H1650 were purchased from ATCC.

### Cell culture and transfection

HEK293T, A549 and H1650 cells were maintained in Dulbecco’s modofied Eagle’s medium (Gibco, 11,965,092) with 10% heat-inactived fetal bovine serum (FBS), 100 units/ml penicillin and streptomycin at 37 °C with 5% CO2. For transfection, the cells were seeded one day before the transfection. The transfection procedures were the same as described previously [13, 14].

### Virus packaging and transduction

The viral packaging was performed as described previously [14, 20]. Briefly, the lentiviral plasmids were co-transfected with psPAX2 (Addgegne) and pMD2.G (Addgene) packaging plasmids into actively growing HEK293KT cells using Lipofectamine 2000 transfection reagent. Viral particle-containing culture medium was collected every 24 h for three times. The medium was cleared by centrifugation at 1000×g for 5 min, and used for infecting target cells in the presence of 6 μg/ml polybrene. The infected cells were selected with puromycin.

### Immunoprecipitation and immunoblotting

Cells were rinsed once with ice-cold PBS and lysed in ice-cold Mammalian lysis buffer (40 mM Hepes (pH 7.4), 100 mM NaCl, 1% Triton X-100, 25 mM glycerol phosphate, 1 mM sodium orthovanadate, 1 mM EDTA, 10 μg/ml aprotinin, and 10 μg/ml leupeptin) or RIPA buffer (40 mM Hepes, pH 7.4, 1% Triton X-100, 0.5% Na-deoxylcholate, 0.1% SDS, 100 mM NaCl, 1 mM EDTA, 25 mM β-glycerolphosphate, 1 mM Na-orthovanadate, 10 μg/ml leupeptin and aprotinin) as indicated. The cell lysates were cleared by centrifugation at 13,000 rpm for 15 min. In immunoprecipitation, primary antibodies were added to the lysates and incubated with rotation at 4 °C for 30 min, followed by adding 20 μl of the protein A-sephorose bead slurry (1:1) into the lysates and incubating with rotation for an additional 3 h. The immunoprecipitates were washed three times with lysis buffer. The cell lysates or immunoprecipitated proteins were denatured by addition of SDS-PAGE sample buffer and boiled for 5 min, resolved by 8%–14% SDS-PAGE. The proteins in the gel were transferred to PVDF membranes (millopore). The immunoblot with chemo-luminescence was performed as described previously [13, 14].

### Immunohistochemistry (IHC)

A tissue microarray containing 63 cases with primary lung adenocarcinoma was used for detection of NEDD4, EGFR and PTEN expression by immunohistochemistry staining. The lung adenocarcinoma tissue samples were collected in Department of Pathology, Affiliated People’s Hospital, Jiangsu University. A single sample was obtained from the center position of each tumor tissue for preparation of the tissue array. All the specimens for this study were obtained with patient informed consent, and the use of these specimens was approved by the Hospital Institutional Review Board. Standard procedure was performed to determine the expression level of NEDD4, EGFR and PTEN in the tumor samples. Immunohistochemical stains were performed on formalin-fixed and paraffin-embedded 4 μm histologic tissue microarray sections. The sections were de-paraffinized and rehydrated in xylene and alcohol bath solution. Antigen unmasking was performed by pretreatment of the slides in 0.01 M citrate buffer (pH 6.0) at 98 °C for 5 min using a microwave oven. The slides were then cooled to room temperature. Endogenous peroxidase was eliminated by incubating the slides in 3% hydrogen peroxide for 10 min. After washed in 10 mM PBS (pH 7.4), the sections were incubated with normal goat serum at room temperature for 10 min, then incubated with a mouse monoclonal antibody to NEDD4 (1:100), a rabbit polyclonal antibody EGFR (1:20), or a monoclonal antibody to PTEN (1:50) at 4 °C overnight. An IHC staining S-P kit (KIT-9710; MAIXIN Biology Corporation, Fuzhou, China) was used to visualize antibody binding on the slides. Counterstaining was performed with hematoxylin. The IHC staining in these specimens was visualized under an Olympus CX31 microscope (Olympus, Center Valley, PA).

### Immunofluorescent staining

The cells were cultured in glass coverslip-bottomed culture dishes (MatTek, Ashland, MA) to 50–80% confluence. After the culture medium was aspirated, the cells were rinsed with PBS twice, fixed with 3.7% paraformaldehyde at 25 °C for 10 min, and permeabilized with 0.2% Triton X-100 in PBS at 25 °C for 10 min. After washing with PBS, the cells were incubated with primary antibody at 8 °C overnight. The cells were washed with PBS three times and incubated with a fluorescent dye-conjugated secondary antibody and phalloidin with at 37 °C for 1–2 h. After washed with PBS three times, fluorescent staining of the cells were visualized under Zeiss LSM710 confocal microscope or Nikon inverted fluorescent microscope.

### Cell migration assays

Cell migration was determined by the wound healing assay and the transwell assay. **(i) The wound-healing assay.** 8X10^5^ cells were seeded on 6-well plates in DMEM supplemented with 10% FBS. 16 h later, the cells reached to about 80–90% confluence in a monolayer. A pipette tip was used to make a straight scratch line in the cell monolayer. The cells were incubated for indicated times and treated as required. The area covered by the migrated cells was quantified with Image J software (from NIH) and used for evaluation of migration rate. **(ii) The transwell assay.** Cells grown in DMEM with 10% FBS were trypsinized and resuspended in DMEM with 10% FBS. 4 × 10^4^ cells were gently added to the upper compartment of Transwell (Corning). DMEM with 10% FBS or EGF were added to the lower compartment of Transwell. The cells were incubated in the culture incubator at 37 °C plus 5% CO_2_ for indicated time. The remained cells on the upper side were gently removed with cotton balls. The cells migrated from the upper side to the lower side through the filter were fixed by 5% glutaraldehyde for 10 min, then stained with 1% Crystal Violet in 2% ethanol for 20 min. The stained cells on the lower side were counted under microscope from 5 different randomly selected views. The cell number averaged from the 5 microscopic views was used as the migration cell number. The migration experiments were repeated three times.

### Quantification of extracellular cathepsin B by ELISA

Extracellular cathepsin B in culture medium was quantified using a human cathepsin B ELISA kit from RayBiotech. Briefly, 50 μl of standards, controls, or diluted culture medium (10 μL culture medium + 40 μL Optimized Assay and calibrator diluents) were added to each well of the ELISA plate, followed by adding100μL of human HRP-conjugated cathepsin B antibody to each well. The assay mix was incubated for 1 h at 37 °C. The mix was aspirated and the well washed 5 times with 1X washing buffer provided in the kit. The substrate solution A (50 μl) and the substrate solution B (50 μl) were added to each well and incubated for 15 min at 37 °C in dark, followed by adding the stop solution (50 μl) to each well. Within 15 min after adding the stop solution, the O.D. absorbance at 450 nm of each was measured using a microplate reader. The amount of cathepsin B in culture medium was calculated from the absorbance using the standard sample plot.

### Statistical analysis of experimental data

The Student t test was used in statistical analysis of experimental data for pair comparison. The *p* value less than 0.05 was considered as statistically significant.

## Abbreviations

ACK1: activated CDC42-associated kinase 1
CXCR2: CXC motif chemokine receptor 2
EGFR: epidermal growth factor receptor
ESCRT: endosomal sorting complex required for transport
HECT: Homologous to the E6-AP Carboxyl Terminus
MVB: multivescular body
NEDD4: neural precursor cell expressed, developmentally down-regulated 4
NEDD4-LD: NEDD4 ligase-dead
PI3K: phosphatidyl inositol-3 kinase
PTEN: Phosphatase and tensin homolog
